# Neutralizing Anti-IL20 Antibody Treatment Significantly Modulates Low Grade Inflammation without Affecting HbA1c in Type 2 Diabetic db/db Mice

**DOI:** 10.1371/journal.pone.0131306

**Published:** 2015-07-10

**Authors:** Christopher Mayer, Regine Bergholdt, Helena Cucak, Bidda Charlotte Rolin, Anette Sams, Alexander Rosendahl

**Affiliations:** 1 Department of Diabetic Complications Biology, Global Research, NovoNordisk A/S, Måløv, Denmark; 2 Department of Translational Pharmacology, Global Research, NovoNordisk A/S, Måløv, Denmark; Emory University, UNITED STATES

## Abstract

Low grade inflammation is present in pre-clinical and human type 2 diabetes. In this process, several cytokines like IL-1β and inflammatory cells like macrophages are activated and demonstrated to participate to the disease initiation and progression. IL-20 is a cytokine known to play non-redundant roles in progression of several inflammatory diseases. To address the therapeutic effect of inhibiting the IL-20 pathway in diabetes, diabetic db/db mice were treated with neutralizing anti-IL20 antibodies *in vivo* and both metabolic and inflammatory parameters were followed. Diabetic islets expressed the IL-20 cytokine and all IL-20 receptor components in elevated levels compared to resting non-diabetic islets. Islets were responsive to *ex vivo* IL-20 stimulation measured as SOCS induction and KC and IL-6 production. Neutralizing anti-IL20 treatment *in vivo* had no effect on HbA1c or weight although the slope of blood glucose increase was lowered. In contrast, anti-IL20 treatment significantly reduced the systemic low-grade inflammation and modulated the local pancreatic immunity. Significant reduction of the systemic IL-1β and MCP-1 was demonstrated upon anti-IL20 treatment which was orchestrated with a reduced RANTES, IL-16 and IL-2 but increased TIMP-1, MCP-1 and IL-6 protein expression locally in the pancreas. Interestingly, anti-IL20 treatment induced an expansion of the myeloid suppressor CD11bGr1^int^ macrophage while reducing the number of CD8 T cells. Taken together, anti-IL20 treatment showed moderate effects on metabolic parameters, but significantly altered the low grade local and systemic inflammation. Hence, future combination therapies with anti-IL20 may provide beneficial therapeutic effects in type 2 diabetes through a reduction of inflammation.

## Introduction

The prevalence of type 2 diabetes (T2D) is estimated to grow globally from 285 million cases in 2010 to 450 million people in 2030 [1]. T2D is associated with increased weight and a state of obesity. In obesity, low grade inflammation associated with activation of immune cells due to various exogenous and endogenous factors is present [2]. Peripheral insulin resistance in adipose tissue is associated with a recruitment of macrophages which participates in pro-inflammatory responses and apoptosis of adipocytes forming crown like structures [3]. The enhanced immune cell accumulation in the adipose tissue leads to enhanced local production of pro-inflammatory cytokines. In T2D, these adipose macrophages constitutes one of the major sources of the enhanced levels of the systemic cytokines [4]. TNFα influences the glucose and lipid metabolism, inhibits insulin action and pancreatic β-cell function and triggers and augments acute and chronic inflammatory processes [5].

Langerhans islets show an accumulation of leukocytes, predominately macrophages [6]. These immune cells show an activated phenotype characterized by enhanced levels of MHCII, galectin-3 and are M1-like polarized based on enhanced expression levels of CD11c [6]. This M1-like macrophage subset is associated with enhanced capacity to produce pro-inflammatory cytokines [7]. Furthermore, elevated glucose activates β-cells directly to release IL-1β [8]. Exposure of β-cells to pro-inflammatory cytokines *in vitro* induces a reduction of insulin production per cell and apoptosis of the β-cells [9–11]. The intricate balance and regulation of IL-1β is termed the inflammasome and involved the regulation of the biological activity of the IL-1β family through caspase-1 activity regulation [12]. This process is described to occur in the T2D islets and to contribute to the disease progression. In pre-clinical experiments of T2D, inhibitors to the IL-1β pathway has been shown to provide some beneficial effects such as recovered β-cell function and improved glucose control although no clinical trials has provided evidence that a stand-alone anti-inflammatory treatment will be efficacious in T2D management [13,14].

IL-20 is a cytokine belonging to the IL-10 family of cytokines which is primarily produced by activated keratinocytes and monocytes [15]. It signals through interactions with a receptor heterodimer complex of IL-20RA/IL-20RB or IL-20RB/IL-22R which is expressed on cells belonging to the epithelial origin [16]. Upon receptor activation, IL-20 phosphorylates STAT3 which regulates proliferation, differentiation of cells and provides a general enhanced pro-inflammatory cytokine signature [17]. Over-activity of IL-20 has been demonstrated in inflammatory conditions of the skin like psoriasis and rheumatoid arthritis [17]. In these diseases, IL-1β and TNFα has also been implemented to play a role in initiation and progression of the disease [6,18].

With the recent understanding that T2D should be considered as an auto-inflammatory disease with low grade inflammation as a hallmark, we evaluated the importance of the IL-20 axis in the pre-clinical spontaneous heterogenic db/db mouse model of T2D using unique neutralizing anti-IL20 antibodies *in vivo*. Our data demonstrate that both the IL-20 cytokine and all the IL-20 receptors are present and functional in the diabetic pancreatic β-cells in the mouse. Inhibition with IL-20 antibodies does not provide a significant effect on HbA1c levels, but does provide a clear reduction of the systemic pro-inflammatory cytokine signature particularly by reduction of IL-1β, reduce systemic CD8 T cells while increasing myeloid suppressor cells and modulates the pancreatic protein composition.

## Material & Methods

### Ethics Statement

All animal experiments were approved by the Copenhagen Animal Ethics Committee and performed according to their recommendations.

### Cell culture

Mouse MIN6 pancreatic β-cells (AddexBio Inc) were cultured in DMEM (25 mM glucose, 2 mM l-glutamine, and 1 mM sodium pyruvate) supplemented with 10% FBS and 100 μM β-mercaptoethanol at 37°C and 5% CO2.

### Isolation of pancreatic islets

Islets of Langerhans were isolated as described previously [6]. Briefly, islets were hand-picked from cold collagenase digested pancreas after repeated thermos shaking at 200 strokes/min washing in HBSS and filtration through a 400 μM cell strainer.

### Gene expression analysis

RNA was isolated from the treated Min6 β-cells and mouse islets or untreated db/db mouse islets using the RNeasy kit with the QiaShredder column (Qiagen, Denmark) following the manufacturer’s protocol. RNA was reverse transcribed to cDNA using cDNA Archive kit (Life Technologies, Denmark) according to the manufacturer’s protocol. qRT-PCR was performed on the Applied Biosystems Prism 7900HT real-time PCR machine and analyzed using SDS 2.4 software (Life Technologies, Denmark). The following primer/probes were purchased from Life Technologies: IL20 (Mm00445341_m1), IL20Ra (Mm00555504_m1), IL20Rb (Mm01232398-m1), IL22R (Mm00663697_m1), SOCS3 (Mm00545913_s1) and Rn18S (Hs99999901_s1). Relative transcript quantities were calculated by the standard curve method and normalized to the reference gene Rn18S.

### Cytokine production from islets

The isolated islets were rested overnight in complete RPMI media and cultured in 96-well plates at concentration of 10 islets/well in presence of increasing concentrations of recombinant IL-20 ng/ml). Supernatants from IL-20 stimulated islets were analyzed on the Bio-Plex 200 (Biorad) using the Milliplex map kit (Millipore) according to the manufacturer’s description.

### *In vivo* evaluation of anti-IL20 effect in db/db mice

Male C57BL/KS db/db mice were obtained from Taconic (Denmark) at the age of 7 weeks acclimatized for one week before start of the experiment. The IgG4 1400-250-5B7 anti-human IL-20 antibody previously that show cross-reactivity to mouse IL-20 (American College of Rheumatology/Association of Rheumatology Health Professionals Annual Scientific Meeting 2012) or vehicle were injected once weekly, day 1, 8 and 15 at a concentration of 20mg/kg i.p with a volume of 5ml/kg. At day 22 the animals were terminated by cervical dislocation in isoflurane.

### End oral glucose tolerance test (OGTT)

At day 21, mice were fasted for 6 h before the OGTT. Mice received 2 g/kg of glucose (200 mg/ml) by oral gavage 10 ml/kg. Blood samples for measurement of blood glucose and p-insulin (marked in bold) were taken at 0 min (i.e. before glucose challenge), 30 min, 60min and 120 minutes post challenge. At day 21, mice were fasted for 6 h before the OGTT. Mice received 2g/kg of glucose (200 mg/ml) by oral gavage 10 ml/kg. Blood samples for measurement of blood glucose and p-insulin were taken at before and at 30 min, 60min and 120 minutes post challenge.

### Blood glucose measurement

Blood glucose was measured twice / week in 10 μl full blood sample taken from the tip of the tail by puncturing the capillary bed with a lancet, using a 10 μl heparinized capillary tube to sample the blood. The blood was then shaken into glucose/lactate System Solution and measured in a Biosen S_Line, autoanalyser (EKF Diagnostics GmbH, Germany) according to the manufacturer’s instructions.

### HbA1c measurement

HbA1c was measured once weekly in 5 ul full blood sample taken from the tip of the tail by puncturing the capillary bed with a lancet, using a heparinized capillary tube to sample the blood. The blood was shaken into 500 μl Hitachi Hemolyzing Reagent and measured in a Hitachi 912 autoanalyser (Roche A/S Diagnostics, Germany) according to the manufacturer’s instructions.

### Plasma insulin measurement

Insulin levels were determined in blood by the luminescence oxygen channeling immunoassay (LOCI) [19]. Briefly, blood taken in capillary tubes was centrifuged in haematokrit centrifuge. 10 μl plasma was transferred directly to micronic tubes. Detection of insulin was by luminescence oxygen channeling immunoassay (LOCI). Anti-insulin mAb RDI-TRK2IP10-D6C4 was conjugated to LOCI acceptor beads (PerkinElmer) and another anti-insulin mAb RDI-TRK2IP10-D3E7 (binding to a different epitope) was biotinylated. The assay was conducted in 384 well plates by adding 1 μl of calibrator, control and unknown sample in the wells followed by 15 μl of a mixture of acceptor beads and biotinylated antibody. After 1 h of incubation at 21–22°C, 30 μl of streptavidin-coated donor beads were added and the plates were further incubated for 30 min. The plates were read in an Envision plate reader (PerkinElmer) at 21–22°C, applying a 520–645 nm filter after excitation by a 680 nm laser. The total measurement time per well was 210 ms including a 70 ms excitation time. During the assay the three reactants combine with analyte to form a bead-aggregate-immunecomplex. Illumination of the complex triggers chemiluminescence from the acceptor beads which is measured in the EnVision plate reader. The amount of light generated is proportional to the concentration of insulin. The concentration of samples was calculated against a standard curve of rat insulin using a 5 parameter fit. The lower limit of quantification was 0.36 ng/ml.

### Expression of inflammatory proteins in the pancreatic tissue

Pancreatic protein lysate was obtained from frozen homogenized pancreatic tissue in presence of protease inhibitors (10 μg/mL Aprotinin, 10 μg/mL Leupeptin, and 10 μg/mL Pepstatin) and 1% Triton X-100. Cellular debris was removed by centrifugation. Eqimolar protein levels were added to the membranes and protein array profiler was then performed according to manufacturer’s description (R&D Systems). Relative expression was determined using Image J software.

### Plasma cytokine analysis

The mice were anaesthetized with isoflurane and blood drawn from the heart into EDTA coated tubes, centrifuged and plasma pipetted into micronic tubes and frozen at -20 C. Levels of IL-1β, TNF-α, IL-6, MCP-1, KC, IL-17 and CXCL10 was determined by Bio-Plex 200 (Bio-Rad, Hercules, CA, USA) using the Milliplex MAP kit (mouse cytokine/chemokine magnetic bead panel; EMD Millipore, Billerica, MA, USA), according to the manufacturer’s description.

### Flow cytometry analysis of splenocyte

Flow cytometric analysis was performed according to standard procedures. Briefly, mice were anaesthetized with isoflurane, terminated by cervical dislocation and spleen removed. Single cells suspensions were obtained after spleens were mashed through a 70um cell strainer and erythrocytes were lysed with cell lysis buffer. Splenocytes were then first blocked for unspecific binding with anti-CD16/CD32 (BD PharMingen, San Diego, CA, USA), followed by surface staining of CD19, CD4, CD8, CD11b and Gr-1 all from BD PharMingen.

Samples were then acquired on a FACS LSRFortessa, equipped with blue, red, and violet laser, followed by data analysis using FACSDiva software (BD Biosciences, San Jose, CA, USA).

### Graphs and statistics

Data are presented as mean ± SEM and were analyzed by one-way ANOVA with Dunnett’s post hoc test for comparisons to the diabetic control group. Analyses were performed using GraphPad Prism for Windows version 6.04.

## Results

### IL-20 is expressed in diabetic db/db islets and contributes to inflammation

In order to determine if the IL-20 axis was present and regulated in islets of Langerhans, the expression of IL-20 and IL-20Rs was evaluated by qPCR. Receptor functionality was monitored by SOCS3 expression and production of pro-inflammatory cytokines.

**Fig 1:**
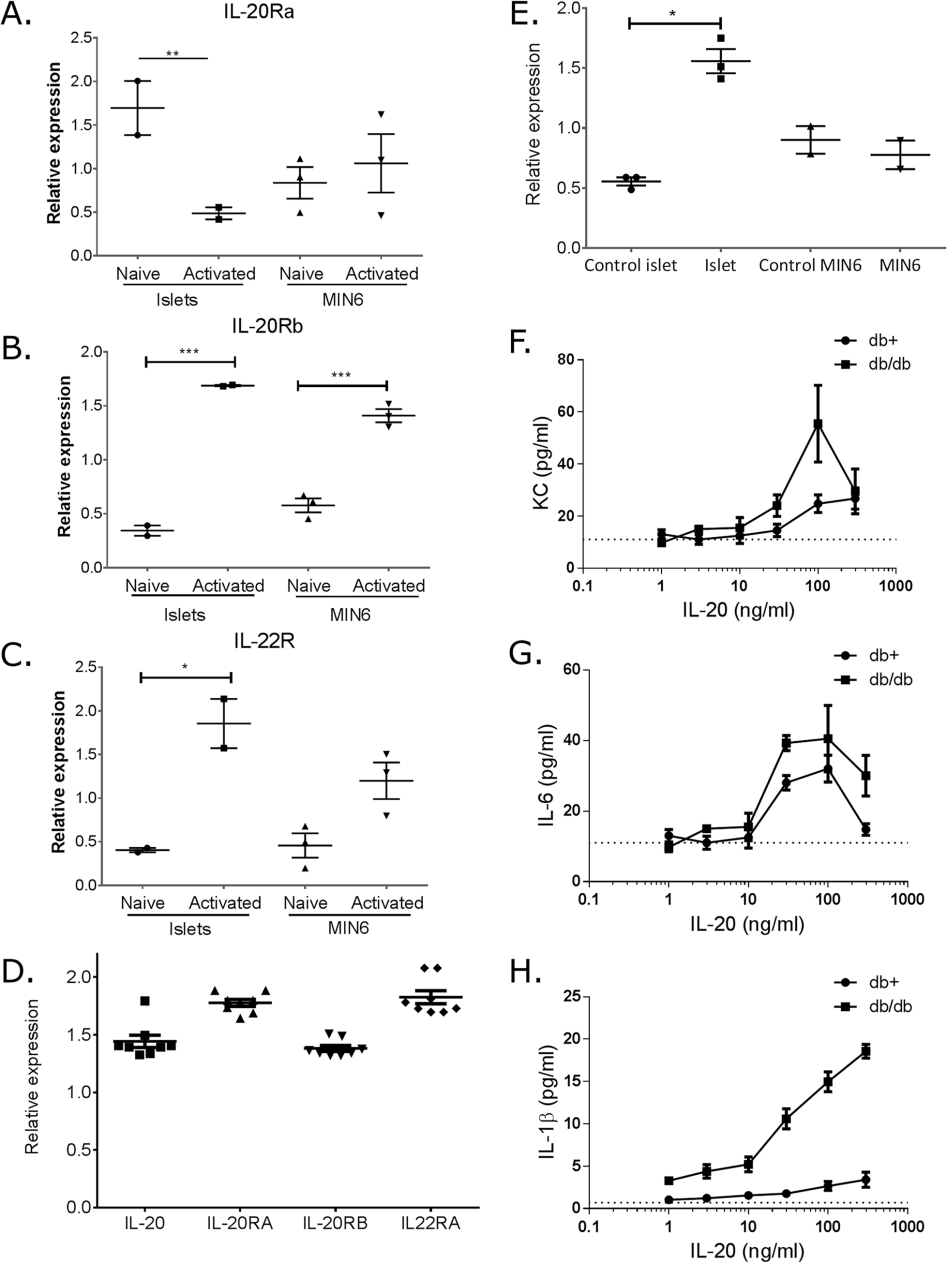
mRNA expression of IL-20 receptors and IL-20 and functional evaluation of IL-20 in murine islets and β-cells. qPCR evaluation of mRNA levels for IL-20RA (A), IL-20RB (B) and IL-22R (C) was evaluated in healthy murine islets and MIN6 β-cells. qPCR evaluation of mRNA levels for IL-20, IL-20RA, IL-20RB and IL-22R was determined in isolated diabetic db/db islets (D). Receptor signaling was monitored in isolated murine islets and β-cells upon activation with IL-20 by measurement of SOCS3 (E). IL-20 induced KC (F), IL-6 (G) and IL-1β (H) was determined in murine islets. To obtain sufficient material, islets from 20 mice were pooled in each experiment (each dot) and all experiments are shown in the representative graphs. Statistical evaluation was performed with 1-way ANOVA.

Healthy islets express all IL-20 receptor chains on mRNA level (Fig 1A–1C). Cytokine activation of islets, significantly down-regulated the IL-20RA while it up-regulated both the IL-20RB and the IL-22R expression (Fig 1A–1C). To determine if the expression was on the β-cells or another cells present within the islets, IL-20R expression was evaluated in the β-cell line MIN6. Similar to the islets, all IL-20 receptor chains were present on MIN6 cells (Fig 1A–1C). In contrast to islets, MIN6 cells showed no regulation of IL-20RA or IL-22R in response to inflammatory cytokines (Fig 1A and 1C). A significant up-regulation of IL-20RB was noted in cytokine activated MIN6 cells (Fig 1B). In the diabetic db/db islets, a clear expression of IL-20 and all IL-20Rs chains were shown to be present (Fig 1D). In fact, the relative expression of IL-20RB and IL-22RA corresponded to the levels obtained in cytokine activated non-diabetic islets (Fig 1A–1D).

Activation with IL-20 for 30 min up-regulated SOCS3 mRNA in murine islets, but not in MIN6 cells (Fig 1E). Healthy islets stimulated with IL-20 weakly induced KC and IL-6, but not IL-1β (Fig 1F–1H). In contrast, diabetic islets responded much more rigorous to IL-20 stimulation with markedly higher production of KC and IL-6 as well as a pronounced induction of IL-1β (Fig 1F–1H). The potency of IL-20 was the same in both diabetic and healthy islets with an estimated EC_50_ for KC at 50 ng/ml, IL-6 at 20 ng/ml and IL-1β at 100ng/ml.

These data demonstrate the regulation of IL-20Rs to be dependent on local inflammation and positively regulated by diabetes. Moreover, the data places the IL-20 and functional IL-20Rs in the micro environment of the diabetic Langerhans islet in the db/db mouse.

### Neutralizing anti-IL20 antibody treatment fails to modulate HbA1c in db/db mice

Based on the *ex vivo* functional support for IL-20 in islets, the effect on metabolic parameters was evaluated in the diabetic db/db model upon neutralizing with anti-IL20 antibody administration.

**Fig 2:**
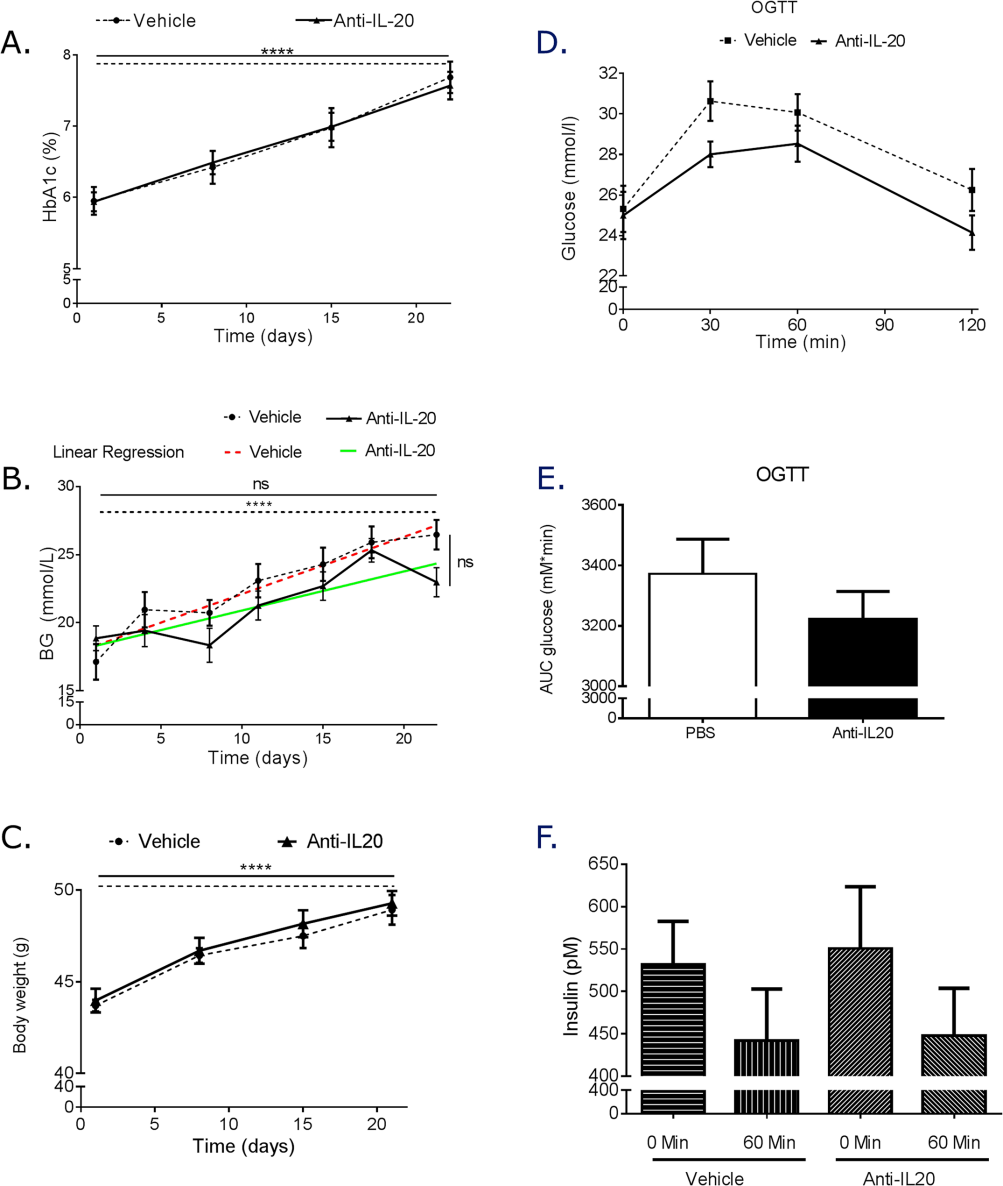
Metabolic effect and oral glucose test after anti-IL20 treatment. One week post last treatment with anti-IL-20, the mice was terminated. During the experiment and at time of termination HbA1c (A), blood glucose (B) and weight (C) was determined. Oral glucose evaluation was conducted one day before termination of the experiment (D). Area under the curve oral glucose test was calculated (E). Plasma level of insulin was measured during the oral glucose tolerance test (F). Each group contained 12 animals. Statistical evaluation comparing the start value in each group with the termination value as well as the termination value between the groups was performed with one way ANOVA in A-C. Linear regression analysis was performed to determine if the two groups showed a difference in the slope of the parameter evaluated during the time of the experiment A-C. Student’s T-test was used to determine statistical significance in E.

HbA1c was progressively increased similarly in both vehicle and anti-IL20 treated animals (Fig 2A). When blood glucose levels were monitored, the vehicle treated mice however showed a highly significant progressive increase measured as significantly higher blood glucose levels at the end of the study compared to at the beginning of the study (Fig 2B). However, the anti-IL20 treated mice although showing elevated levels at the end of the treatment period did not show a significant increase compared to the start values (Fig 2B). Despite this difference in slopes (vehicle: 0.4202 mmol/L / day ± 0.06327 versus anti-IL20 treated: 0.2876 mmol/L / day ± 0.05928), there was no significant reduction of blood glucose levels at the end of the study period between the vehicle and the anti-IL20 treated animals. Neutralizing anti-IL20 treatment had no effect on weight gain (Fig 2C).

With the potential effects on blood glucose increase rate by anti-IL20 treatment, an oral glucose tolerance test was performed and the insulin levels were measured. Although levels of glucose were lower in the anti-IL20 treated animals during the oral glucose evaluation, the effect did not reach significance during the 120 minute test (Fig 2D and 2E). The plasma level of insulin was also shown to be similar in the vehicle treated group and in the anti-IL20 treated group (Fig 2F).

These data demonstrate that anti-IL20 treatment for 3 weeks had no effect on HbAc1 or weight gain but did reduce the slope of blood glucose increase. Anti-IL20 did not improve OGTT or modulated the circulating insulin levels.

### Neutralizing anti-IL20 antibody treatment significantly reduces the systemic low grade inflammation in diabetic mice

To evaluate if neutralizing anti-IL20 antibody treatment reduced the low grade cytokine signature present in T2D, plasma cytokine levels was determined after treatment.

**Fig 3:**
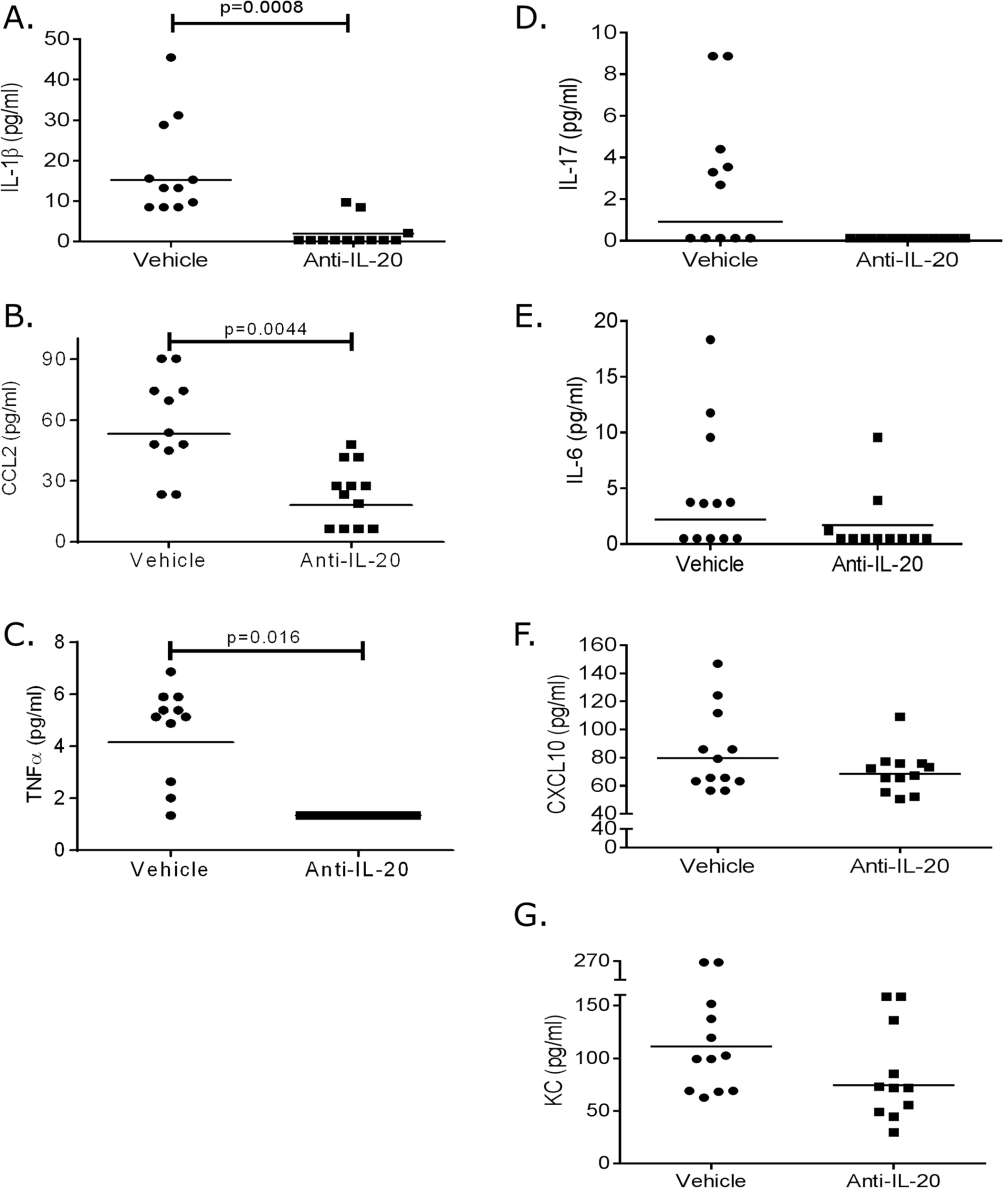
Plasma cytokine signature. At termination of the experiment, plasma was generated from the mice and analyzed for IL-1β (A), CCL2 (B), TNFα (C), IL-17 (D), IL-6 (E), CXCL10 (F) and KC (G). Graph shows individual values from each mouse (each group had 12 mice) and the corresponding geometic mean value. Statistical evaluation is performed with 1-way ANOVA.

Vehicle treated animals showed low heterogeneous expression of several pro-inflammatory cytokines in the plasma (Fig 3A–3G). Treatment with neutralizing anti-IL20 significantly reduced the T2D associated IL-1β and MCP-1 cytokines (Fig 3A and 3B). Furthermore, anti-IL20 treatment interestingly reduced both TNFα and IL-17 to levels below detection limit (Fig 3C and 3D). The effect of IL-6, CXCL10 and KC followed the same trend, but did not reach significance (Fig 3E–3G).

Taken together, these data demonstrate that neutralization of IL-20 reduced the systemic low grade inflammation thereby suggesting that IL-20 directly contributes to the pro-inflammatory signature noted in T2D.

### Neutralizing anti-IL20 antibody treatment modulates the systemic lymphoid composition in the spleen

With the therapeutic effect obtained on systemic cytokines after neutralization of IL-20, the effect on the systemic immune cell compartment was determined in the spleen as this tissue previously has been shown to reflect systemic immune modulations distinguishing healthy from diabetic mice [6].

**Fig 4:**
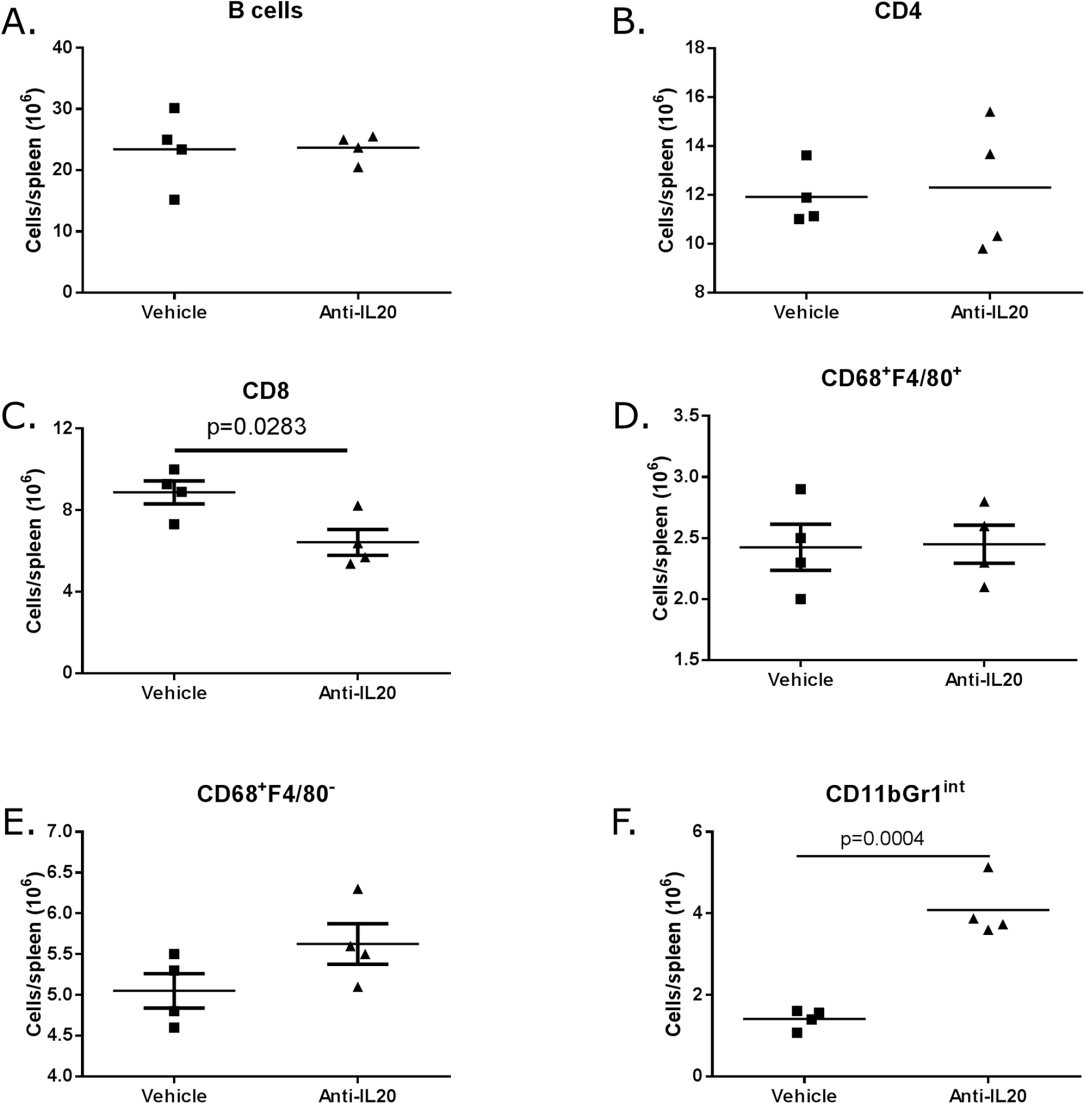
Flow cytometric evaluation of spleen after treatment with anti-IL20. At termination of the experiment, the spleen was removed and presence of B cells (A), CD8 T cells (B), CD4 T cells (C) and CD11bGr1int macrophages (D) was determined by flow cytometric analysis. Each group had four spleen evaluated. Statistical evaluation is performed with 1-way ANOVA.

Anti-IL20 treatment had no effect on the presence of B cells or CD4^+^ T cells in the spleen (Fig 4A and 4B). However, only three weeks of treatment with anti-IL20 significantly reduced the number of CD8 T cells in the spleen (Fig 4C). Although not statistically modulated, the CD68^+^F4/80^-^ macrophage sub-population increased in number while the CD68^+^F4/80^+^ macrophage sub-population was unaffected by anti-IL20 treatment (Fig 4D and 4E). Most interestingly, the number of myeloid suppressor CD11bGr1^int^ cells was significantly enhanced in the spleen in response to anti-IL20 treatment (Fig 4F).

Taken together, these results show that neutralization of IL-20 modulated the systemic immune cell composition of inflammatory cells in the spleen characterized by a reduction of CD8 T cells and an increase of macrophages expressing CD11bGr1^int^.

### Neutralizing anti-IL20 antibody treatment influences the expression of inflammatory and tissue remodeling components in the pancreatic tissue

In order to determine if anti-IL20 treatment modulated the local composition of pro-inflammatory cytokines and remodeling proteins a protein array study was evaluated on the whole pancreatic tissue.

**Fig 5:**
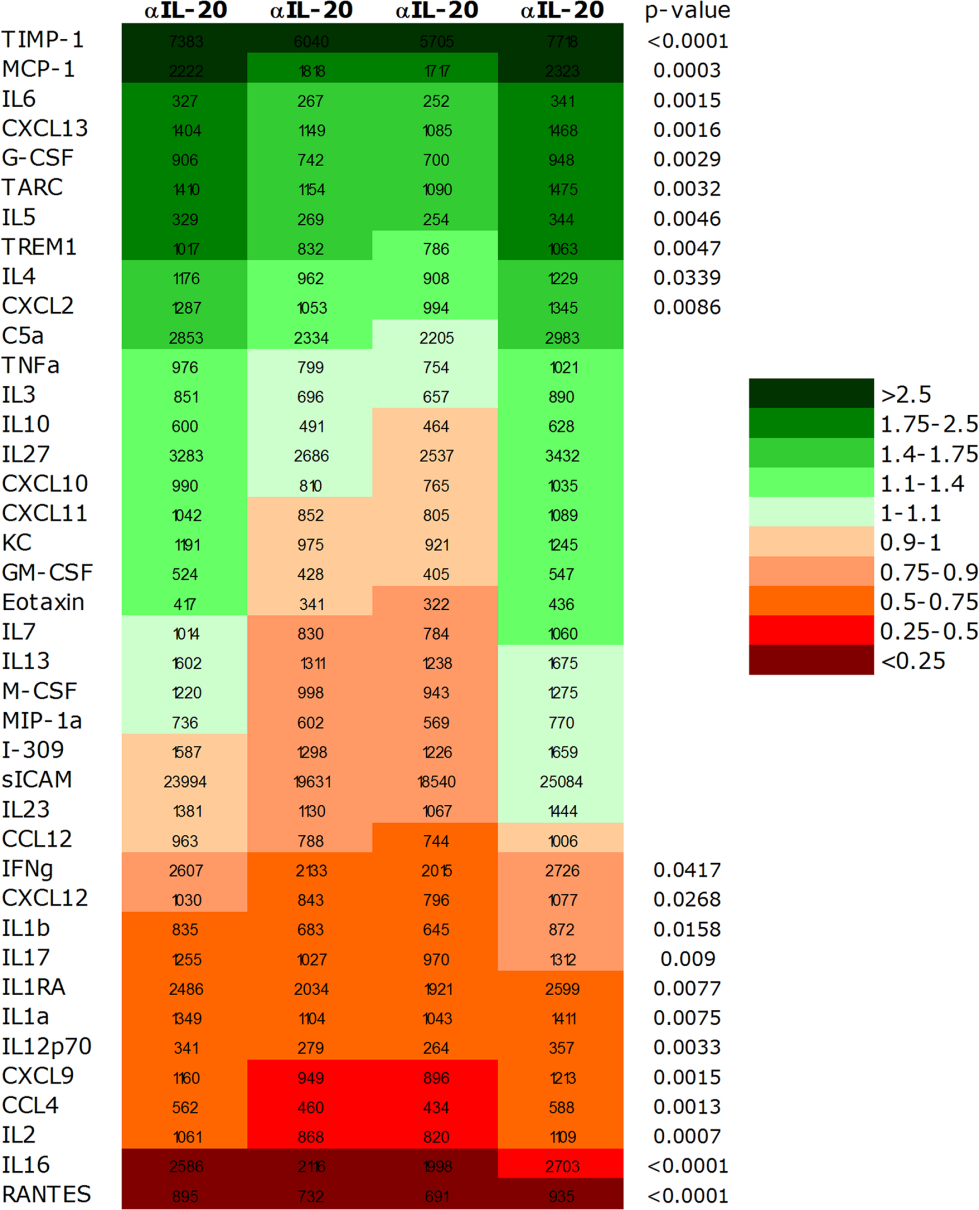
Heat map analysis and the relative expression of proteins in pancreas at the termination of the experiment. Heat map analysis showing the proteins up (green) and down (red) regulated by anti-IL20 treatment arranged in order of fold modulation (increased on top and decreased on bottom) compared for individual anti-IL20 antibody treated mice compared to vehicle treated mice. Four pancreatic tissues were evaluated in each group. Statistical evaluation is performed with 1-way ANOVA.

**Fig 6:**
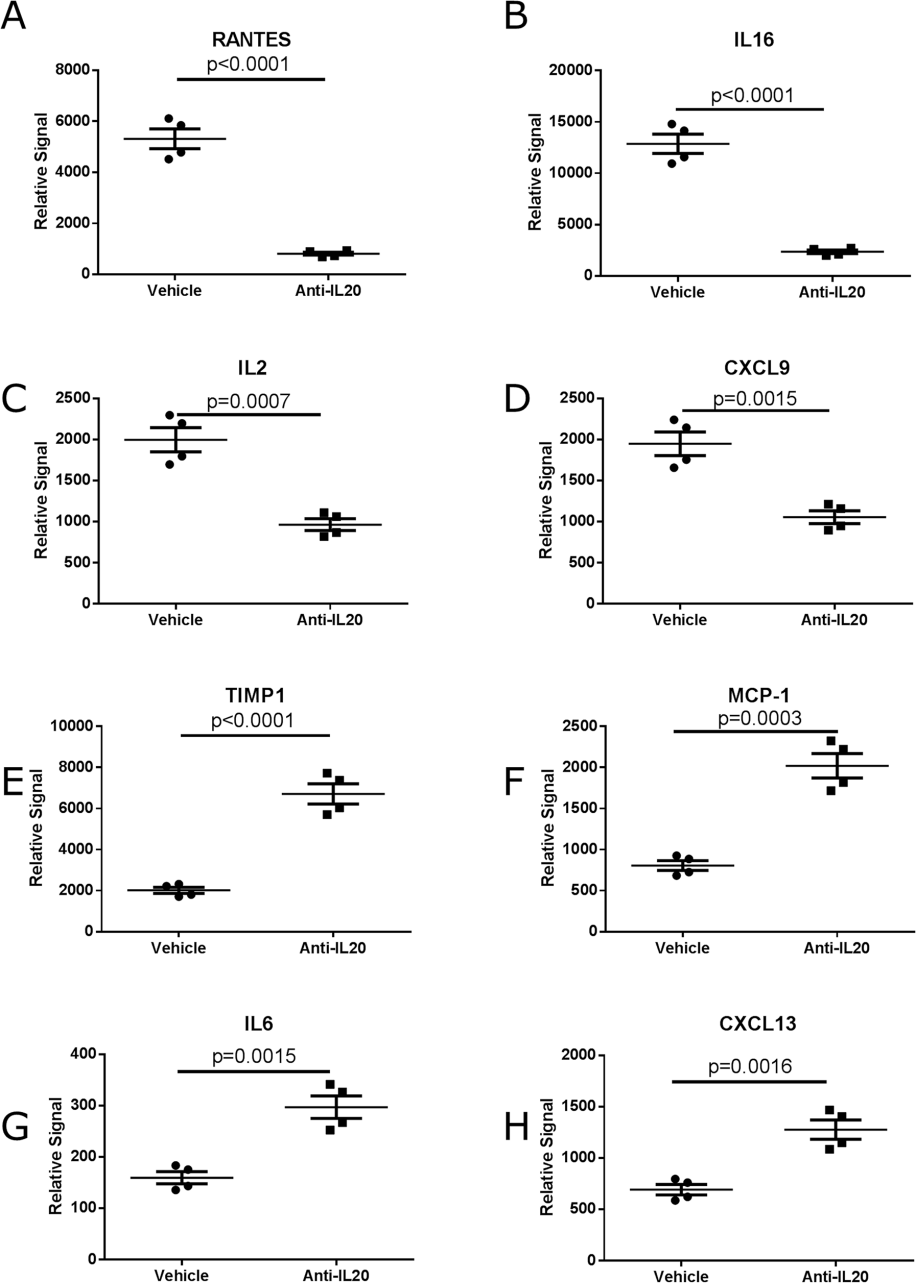
Proteins regulated in the diabetic pancreatic tissue after anti-IL20 antibody treatment compared to vehicle treatment. The proteins demonstrating most pronounced inhibition by anti-IL20 antibody treatment RANTES (A), IL-16 (B), IL-2 (C), CXCL9 (D). The proteins demonstrating most pronounced induction by anti-IL20 antibody treatment TIMP-1 (E), MCP-1 (F), IL-6 (G) and CXCL13 (H). Four pancreatic tissues were evaluated in each group. Statistical evaluation is performed with 1-way ANOVA.

Treatment with anti-IL20 significantly modulated expression of several proteins in the diabetic pancreatic tissue both by induction and by inhibiting the protein levels (Fig 5). In total ten of the investigated proteins were significantly upregulated, while twelve proteins were significantly down-regulated upon anti-IL20 treatment (Fig 5). Of the upregulated proteins chemokines, cytokines and some tissue remodeling factors were noted (Fig 5). The down-regulated proteins were either cytokines or chemokines, but importantly included effector cytokines like IL-1β, IL-12p70 and IL-17 (Fig 5). Most pronounced inhibition was observed on the levels of RANTES (p<0.0001), IL-16 (p<0.0001), IL-2 (p = 0.0007) and CXCL9 (p = 0.0013) (Fig 6A–6D). In contrast, the most pronounced induction by anti-IL20 treatment was demonstrated on TIMP-1 (p<0.0001), MCP-1 (p = 0.0003), IL-6 (p = 0.0015) and CXCL13 (p = 0.0016) (Fig 6E–6H).

Taken together, these data shows that systemic anti-IL20 treatment markedly modulates the local expression of pro-inflammatory and tissue remodeling proteins in the diabetic pancreatic tissue.

## Discussion

The epidemic growth of obesity and T2D emphasize the importance of novel therapies. Recent molecular understanding in both clinical subjects and in pre-clinical models has provided support that inflammation is strongly associated with insulin resistance [20]. Furthermore, in the recent years it has been demonstrated that inflammatory cells, particularly macrophages, accumulate in the T2D Langerhans islet. Invading macrophages poses mechanisms that contribute to local enhanced inflammation leading to functional deficient islet function and ultimately of direct macrophage dependent elimination of the insulin producing β-cells in T2D model [6,21].

Our demonstration that IL-20 and the cognate receptors are present in elevated levels in cytokine activated and T2D islets from db/db mice are hence intriguing and may provide a novel approach to treat T2D disease so far not evaluated. Cytokine mediated apoptosis of β-cells is a well-established phenomenon and is considered as key mechanisms in T2D by reducing the number of insulin producing cells at a stage when an increase is required to compensate for the hyperglycemic state [9,10]. Our observation that whole T2D islets have an augmented dose-dependent responsiveness, characterized by induction of IL-6 and KC, to IL-20 challenge is intriguing as it suggests a functional linkage between IL-20 and the local inflammation observed in the T2D islet [6,9]. KC is a chemokine produced by macrophages present in the islets as well as by epithelial cells while IL-6 is a cytokine known to either promote or modulate other inflammatory responses [22–24]. The observation that only whole islets respond to IL-20 whereas the β-cell line MIN6 is unresponsive suggests that the main responder cell might be the endothelial cells present in the islet which augments the local inflammation in the islet micro environment though IL-20 signaling. Presence and function of IL-20 has previously been described in other endothelial systems that are linked to local inflammation such as atherosclerosis, psoriasis and rheumatoid arthritis [25–28]. There, IL-20 mounts a more rigorous immune response by activating the endothelial cells increasing the permeability allowing immigration of effector immune cells like macrophages [26–28]. The macro vascular complication atherosclerosis is common in T2D subjects and indeed associated with IL-20 activity [27,28]. In micro vasculature complication to diabetes such as diabetic nephropathy endothelial dysfunction such as increased vascular permeability is associated with both glomeruli sclerosis and interstitial fibrosis. This leads to excessive tissue modulations that in a chronic phase lead to enhanced protein in the urine. During the disease progression several inflammatory pathways are activated with specific cytokine signatures and influx of various leukocytes such as macrophage subpopulations. Whether IL-20 plays a similar function in the diabetic islet micro vasculature remains to be established, but the receptor expression and cytokine production implies that this may indeed be the case.

T2D is characterized by a progressive increase in hyperglycemia and peripheral insulin resistance [29]. Treatment with neutralizing anti-IL-20 antibodies did not significantly improve the HbA1c or blood glucose at the end of the study period. However, the rate of blood glucose increase was lower in the anti-IL20 treated animals. In humans the intra-individual variation of HbA1c in non-diabetic subjects with normal glucose levels is reported to be minimal [30] In contrast, several groups have reported that HbA1c values in patients may not be constant among all individuals despite the presence of similar blood glucose or fructosamine concentrations [31]. One potential explanation to this might be that the rate of the glycation of hemoglobin may show intra-individual difference. In fact individuals can be divided into low and high glycators which results in the so call termed glycation gap [32]. Although the db/db mouse model is an inbreed mouse strain, the disease develops endogenously with onset starting individually between mice from week 6 of age until week 10 of age. Furthermore, the severity of T2D varies from moderate to very severe within 6–8 weeks, measured as HbA1c. Thus, the discrepancy between the beneficial effects on the short term blood glucose levels and the lack of long term effects on HbA1c could potentially reflect a heterogeneous population of glycotors within the animals in the study.

The lifespan of the erythrocytes in non-diabetic subjects is approximately 140 days. In contrast, in diabetic subjects the erythrocyte life-span is reduced to 80 days [33]. As the level of HbA1c is a measurement of the glycosylated level of hemoglobulin present at a given time-point, the HbA1c level will increase if the life-span of the erythrocyte is prolonged by the treatment regime due to the inertness in the system. IL-20 does not induce colony formation of CD34^+^ precursor cells, but synergize with colony stimulating factor (CSF) to increase the size of the colonies formed [34]. The colonies formed mainly consisted of erythrocytes and megakaryocytes, but also contained precursors for granulocytes and monocytes. *In vivo*, IL-20 transgenic mice have a moderately enhanced level of erythrocytes [34,35]. Thus, only hypothesis for the discrepancy between the effects on blood glucose and HbA1c by neutralizing anti-IL20 could be that anti-IL-20 treatment potentially influences the level of erythrocytes being generated from precursor cells resulting. This could result in a shift in lifespan of mature erythrocytes which could influence the HbA1c levels differently compared to the more short term blood glucose levels. This potential effect on erythrocyte lifespan by anti-IL20 remains to be determined.

Diabetic subjects and pre-clinical models of T2D display a metabolic syndrome associated with low grade inflammation [6,20,36]. This is associated with enhanced levels of cytokines and influx of inflammatory cells into peripheral tissues [6,37,38]. The majority of the cells invading islets are M1-like macrophages in early disease which over time are repolarized into M2-like macrophage in the systemic compartment that contributes to excessive production of tissue remodeling factors [6,21]. In T2D diabetic db/db mice, anti-IL20 had no effect on B cell and CD4^+^ T cell, but did reduce the number of CD8^+^ T cells. Furthermore, treatment did show an expansion of a specific myeloid suppressor macrophage subtype upon treatment which has previously been suggested to have functions in inflammatory diseases such as asthma and is present in elevated number in cancer patients where they inhibit tumor elimination [39–41]. The regulatory function in this subset of cells has been shown to be dependent on down-regulation of the transcriptional component IRF-8 in a G-CSF dependent manner [41]. Our observation that anti-IL20 treatment significantly increases the expression of G-CSF locally in the pancreatic tissue upon treatment suggests that IL-20 receptor signaling may regulate the production of G-SCF and hence participate in myeloid suppressor cell induction. In fact, in the pancreatic tissue a heterogeneous regulation of several cytokines and chemokines was noted upon anti-IL20 treatment. Several pro-inflammatory cytokines like IL-1α previously demonstrated to participate in the inflammasome activation in β-cells and to participate in development of T2D was significantly reduced by anti-IL20 administration. In contrast, local expression of MCP-1 was significantly induced by anti-IL20 treatment while it showed a significant reduction in the systemic compartment. Herder et al showed that the role of systemic MCP-1 levels in development of T2D remains controversial [42]. TIMP-1 inhibits the activity of several MMPs as well as controls proliferation and apoptosis of a variety of cells types. Anti-IL20 treatment significantly induced protein expression of TIMP-1 locally in the pancreas. Intriguingly, inhibition of the IL-20 axis in carbon tetrachloride induced liver injury reduces diseases severity by restoring hepatocyte proliferation and prevents liver fibrosis by inducing high expression levels of TMP-1 [43]. Moreover, Jiang et al demonstrated that overexpression of TIMP-1 in pancreatic β-cells protects from diabetes induced by low dose administration of streptozotocin by reducing β-cell apoptosis [44].

Taken together, our data shows that the full functional IL-20-axis is present in the diabetic islet where it signals to further enhance the pro-inflammatory signature. Importantly, our data shows that intervention with anti-IL-20 in diabetic mice fails to improve HbA1c, but does modulate the systemic and local inflammatory response. Current therapeutic intervention in the clinic although showing good effect on glycemic control, does not specifically target the low grade inflammation in the patients which often remains even under optimal treatment regime [6,21]. Furthermore, >30% of the diabetic patients progress to develop complications like diabetic nephropathy, which has been shown dependent on inflammatory pathways [45]. Thus, the data suggests that anti-IL-20 treatments might be considered as an add-on therapy to reduce the low grade inflammatory response still present after current first line.
